# Elevated Atmospheric CO_2_ Triggers Compensatory Feeding by Root Herbivores on a C_3_ but Not a C_4_ Grass

**DOI:** 10.1371/journal.pone.0090251

**Published:** 2014-03-20

**Authors:** Scott N. Johnson, Goran Lopaticki, Susan E. Hartley

**Affiliations:** 1 Hawkesbury Institute for the Environment, University of Western Sydney, Penrith, New South Wales, Australia; 2 York Environmental Sustainability Institute, Department of Biology, University of York, York, United Kingdom; Lakehead University, Canada

## Abstract

Predicted increases in atmospheric carbon dioxide (CO_2_) concentrations often reduce nutritional quality for herbivores by increasing the C∶N ratio of plant tissue. This frequently triggers compensatory feeding by aboveground herbivores, whereby they consume more shoot material in an attempt to meet their nutritional needs. Little, however, is known about how root herbivores respond to such changes. Grasslands are particularly vulnerable to root herbivores, which can collectively exceed the mass of mammals grazing aboveground. Here we provide novel evidence for compensatory feeding by a grass root herbivore, *Sericesthis nigrolineata*, under elevated atmospheric CO_2_ (600 µmol mol^−1^) on a C_3_ (*Microlaena stipoides*) but not a C_4_ (*Cymbopogon refractus*) grass species. At ambient CO_2_ (400 µmol mol^−1^) *M. stipoides* roots were 44% higher in nitrogen (N) and 7% lower in carbon (C) concentrations than *C. refractus*, with insects performing better on *M. stipoides*. Elevated CO_2_ decreased N and increased C∶N in *M. stipoides* roots, but had no impact on *C. refractus* roots. Root-feeders displayed compensatory feeding on *M. stipoides* at elevated CO_2_, consuming 118% more tissue than at ambient atmospheric CO_2_. Despite this, root feeder biomass remained depressed by 24%. These results suggest that compensatory feeding under elevated atmospheric CO_2_ may make some grass species particularly vulnerable to attack, potentially leading to future shifts in the community composition of grasslands.

## Introduction

The largest annual increase in global atmospheric CO_2_ emissions in the last 50 years occurred during 2010 [1]. Such increases will impact on ecological communities and the species interactions within them. For example, it is widely observed that elevated atmospheric CO_2_ concentrations (eCO_2_) reduces the nutritional quality of plants for herbivores [2]. A meta-analysis of over 100 published studies demonstrated that while both carbon (C) and nitrogen (N) increased in both roots and shoots, C increased at an accelerated rate relative to N and led to an average increase in C∶N ratios of 11%, effectively reducing nitrogen concentrations in both roots and shoots [3], either by dilution or reallocation [4]. Since N is the limiting factor in most herbivore diets [5], herbivores may respond to this decline in host quality by compensatory feeding, whereby the herbivore eats more plant biomass in an attempt to acquire adequate nutrition [4], [6]. Indeed, Stiling and Cornelissen's [2] meta-analysis reported that relative consumption by insect herbivores increased by 17% and total consumption by 19%, when feeding on plants under eCO_2_. Given that eCO_2_ can cause similar increases in C∶N ratios in the roots as in the shoots [3], it is surprising that feeding responses of root herbivores to such changes in chemistry have not been examined [7]. To our knowledge, only four studies have investigated the effects of eCO_2_ on root feeding insects [8]–[11] and none have investigated this for grasses.

Root herbivores are major components of many ecosystems, having the capacity to shape the community structures of other herbivores and plant communities [12], [13]. Grassland systems can be particularly vulnerable to root herbivores [14]. For instance, in some pasture systems it is not uncommon for the collective biomass of root herbivores to exceed that of grazing mammals aboveground [15]. In addition to covering over 40% of the planet's land surface area [16], grasslands are responsible for storing over one third of global terrestrial carbon stocks [17]. Grasslands often comprise of C_3_ and C_4_ grass species; C_3_ grasses are usually superior hosts for herbivores compared with C_4_ grasses, but they also are more strongly affected by elevated CO_2_, generally showing greater increases in C and reductions in N concentrations than C_4_ plants [18], [19]. This occurs because Rubisco, the initial carboxylating enzyme to facilitate the assimilation of CO_2_ into carbohydrates operates below its maximum capacity at current CO_2_ concentrations in C_3_ plants, so has the greater capacity to respond to eCO_2_ [4], [20]. Because of this, C_3_ plants may be disproportionately subject to compensatory feeding under eCO_2_. This has rarely been tested for aboveground herbivores (e.g. [21], [22]), and never, to our knowledge, for root herbivores.

This study characterised how eCO_2_ affected a C_3_ (*Microlaena stipoides*) and a C_4_ (*Cymbopogon refractus*) grass species, and how any changes in grass traits affected the feeding behaviour and performance of a root feeding insect, the scarab *Sericesthis nigrolineata* Boisduval (Coleoptera: Scarabaeidae). We hypothesised that: (1) *M. stipoides* would be nutritionally superior (higher N, lower C∶N) than *C. refractus* under ambient CO_2_ (aCO_2_), (2) under eCO_2_, both grasses would become inferior hosts for the root herbivore due to lower N and higher C∶N, and this effect would be more pronounced for *M. stipoides* than *C. refractus*; (3) under eCO_2_, insects would consume more root tissue through compensatory feeding, with the biggest increase on *M. stipoides* and (4) scarab performance (body mass) would better on *M. stipoides* than *C. refractus*, but would decline at eCO_2_.

## Materials and Methods

### Chambers

Six glasshouse chambers, three maintained at aCO_2_ of 400 µmol mol^−1^ and the other three at eCO_2_ (600 µmol mol^−1^), were used. These chambers (3 m×5 m×3 m; width×length×height) with UV transparent plexiglass (6 mm thick) walls and roof were naturally lit throughout the experiment. Daytime air temperature was regulated to reach a midday peak of 24°C and fall to 21°C at midnight (±4°C) at night time. Humidity was controlled at 60% (±6%). CO_2_ levels were controlled via the monitoring and control system PlantVisorPRO (Carel Industries, Padova, Italy). Briefly, CO_2_ levels within each chamber were monitored by a CO_2_ probe (GMP222, Vaisala, Vantaa, Finland), with CO_2_ (food grade, AirLiquide, Australia) injected from pressurized cylinders through solenoid valves. Before entering a chamber, CO_2_ was passed through a Purafils column to eliminate possible ethylene contamination.

### Experimental procedure

**Figure 1:**
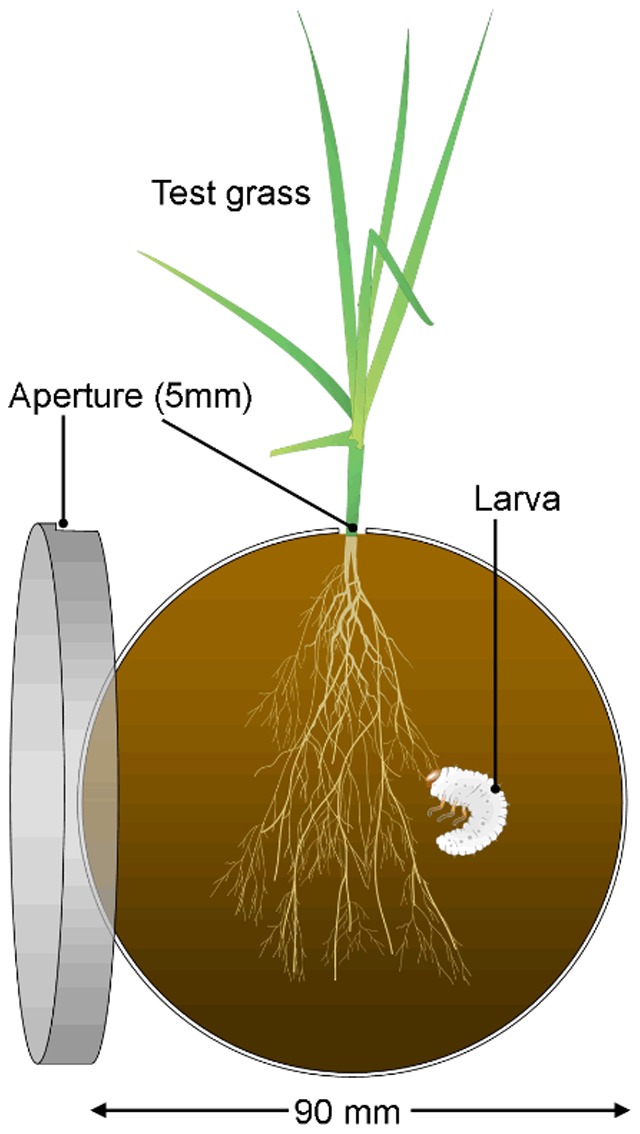
Bioassay cage used to determine root consumption and change in body mass of larval scarab beetles.

Grasses were planted in 90 pots (100 mm diameter) containing 850 g of air-dried and sieved (1 mm) soil, which was loamy-sand with low (0.7%) organic matter (full details given in [23]). These were randomly assigned to the six climate chambers (15 in each) and watered daily to maintain soil water content at 15%, which was verified with a two-rod moisture probe (Hydrosense, Campbell Scientific, Australia). No nutritional supplement was provided. After 10 weeks, five of the plants for each species were selected and shoots and roots were separated, oven dried (40°C) and weighed. For the remaining 10 plants, a single seedling comprising small section of roots and grass blades (*c.* 2–3 g fresh mass) was teased apart and transferred into bioassay cages (Fig. 1) constructed from 90 mm Petri dishes filled with soil (details as above). Cages were a variation of similar bioassay cages used for measuring root damage by root feeding insects in other studies [24], [25]. One side had an aperture, through which the grass blades were left exposed. Dishes were wrapped in tinfoil and stored vertically in the chambers. Moisture was maintained by devlivering 2–3 ml water daily via the aperture. After 3 d, a single second instar larva was weighed and placed inside half of the cages, selected at random. Insects were from an established culture at UWS previously obtained from a site containing a range of C_3_ and C_4_ grasses [26]. After 7 d, the larva was removed and re-weighed. Roots were snap-frozen, freeze-dried and weighed. Material was milled and analysed for C and N concentrations using a LECO TruSpec® CHN analyser.

### Statistical analysis

Analysis of variance (ANOVA) tests, in which chamber (and hence the three replicates of CO_2_ treatment) were included as block terms to avoid pseudo-replication, were used. Plant biomass, root consumption and larval mass was analysed with a two-way ANOVA (grass species and CO_2_, with an interaction of each term) with initial larval mass included as a covariate in the latter case. Chemistry was analysed with three-way ANOVAs (grass species, CO_2_ and insect presence, with interactions of each term). Differences between treatments were determined using least square mean tests when significant interactions between CO_2_ and grass species existed. Unless indicated otherwise all analysis was conducted on untransformed data using Genstat (version 14, VSN International, UK).

## Results

### Plant responses

**Figure 2:**
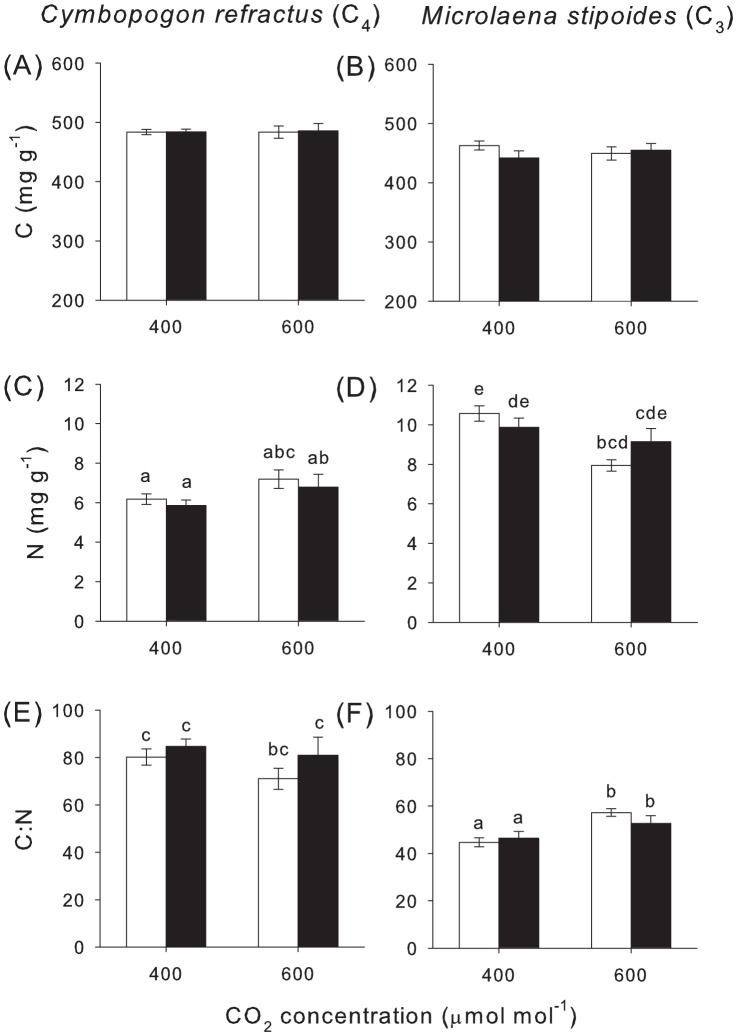
Carbon and nitrogen concentrations in grass roots. (A–B) Carbon (C), (C–D) nitrogen (N) concentrations and (E–F) C∶N ratio in roots of C_3_ (*Microlaena stipoides*) and C_4_ (*Cymbopogon refractus*) grass species with (closed bars) and without (open bars) larval feeding. Mean ± S.E. shown, N = 15. Lowercase superscripts indicate significant differences between treatments.

Plant biomass was unaffected by eCO_2_, for either grass species, although *C. refractus* plants were significantly bigger than *M. stipoides* largely due to higher shoot mass (Table 1). Concentrations of root C were higher in *C. refractus* (Fig. 2A) than *M. stipoides* (Fig. 2B), but largely unaffected by other variables (Table 2). In contrast, root N concentrations were higher in *M. stipoides* than *C. refractus* (Fig. 2 C–D), but in this case there was also a significant interactive effect of CO_2_ and grass species (Table 2). In particular, eCO_2_ reduced root N concentrations in *M. stipoides*, but not *C. refractus* (Fig. 2C–D). Roots of *C. refractus* had a higher C∶N ratio than *M. stipoides* (Fig. 2E–F). Again there was a significant interaction between eCO_2_ and grass type (Table 1); eCO_2_ caused an increase in root C∶N in *M. stipoides* (Fig. 2F), but not in *C. refractus* (Fig. 2E).

**Table 1:** Plant biomass responses to ambient and elevated CO_2_ conditions.

Grass species	Atmospheric CO_2_ concentration	Plant biomass (g)		
		Total	Shoot	Root
*Cymbopogon refractus* (C_4_)	400	3.31±0.23	2.51±0.15	0.79±0.09
	600	2.91±0.19	2.31±0.16	0.59±0.05
*Microlaena stipoides* (C_3_)	400	2.49±0.16	1.81±0.11	0.67±0.07
	600	2.27±0.12	1.67±0.10	0.60±0.05
CO_2_ (F_1,4_)		F = 1.56, *P* = 0.280	F = 1.82, *P* = 0.248	F = 0.85, *P* = 0.409
Grass species (F_1,108_)		**F = 17.79, ** ***P*** **<0.001**	**F = 26.23, ** ***P*** **<0.001**	F = 1.11, *P* = 0.297
CO_2_×grass species (F_1,108_)		F = 0.31, *P* = 0.582	F = 0.05, *P* = 0.822	F = 1.42, *P* = 0.239

Statistically significant effects indicated in **bold.**

**Table 2:** Summary of statistical analysis for carbon and nitrogen concentrations.

Responses	Fixed effects													
	CO_2_		Insects		CO_2_×Insects		Grass species		CO_2_×Grass species		Grass species×Insects		CO_2_×Grass species×Insects	
	F_1,4_	*P*	F_1,108_	*P*	F_1,108_	*P*	F_1,108_	*P*	F_1,108_	*P*	F_1,108_	*P*	F_1,108_	*P*
Root Carbon^1^ – Fig. 1A–B	0.01	0.962	0.25	0.617	1.09	0.299	**23.28**	**<0.001**	0.01	0.922	0.48	0.490	0.83	0.363
Root Nitrogen^1^- Fig. 1C–D	0.43	0.547	0.17	0.680	1.53	0.219	**89.91**	**<0.001**	**17.08**	**<0.001**	1.20	0.276	2.35	0.129
C∶N – Fig. 1E–F	0.23	0.653	1.09	0.299	0.01	0.933	**109.62**	**<0.001**	**8.15**	**0.005**	2.39	0.125	1.15	0.286

Statistically significant effects indicated in **bold**.

1 Arcsine square root transformation applied prior to analysis.

### Insect responses

**Figure 3:**
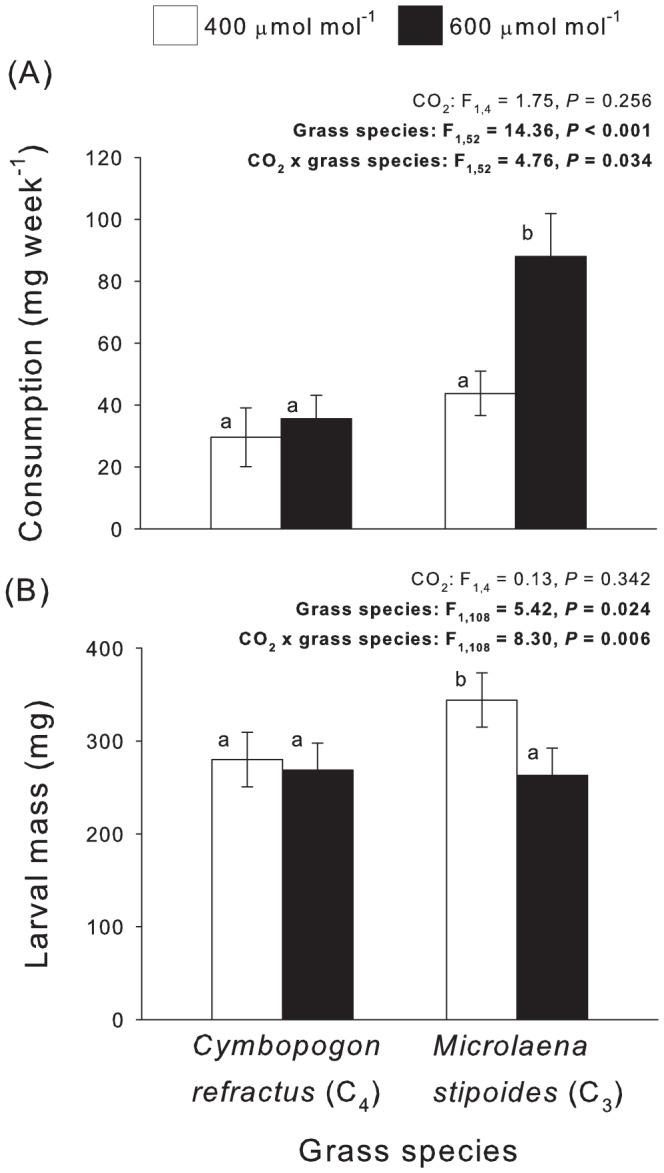
Feeding and performance (body mass) of larvae. (A) Root consumption and (B) larval mass when reared on *M. stipoides* and *C. refractus* under ambient and elevated CO_2_. Mean ± S.E. shown, N = 15. Lowercase superscripts indicate significant differences between treatments. Statistically significant terms indicated in bold.

CO_2_ did not affect root consumption overall (Fig. 3A) but consumption rose significantly on *M. stipoides* under eCO_2_. No difference in root consumption was seen on *C. refractus* under either CO_2_ treatment (Fig. 3A). The final mass of larvae was higher on *M. stipoides* than *C. refractus* at aCO_2_, but under eCO_2_ conditions larval mass was reduced to levels seen for those feeding on *C. refractus* (Fig. 3B).

## Discussion

This study demonstrates that eCO_2_ negatively affects a grass root herbivore when feeding on *M. stipoides*, a C_3_ grass, but not on *C. refractus*, a C_4_ grass. Elevated CO_2_ caused bigger reductions in *M. stipoides* quality than in *C. refractus*, lowering N concentrations and increasing the C∶N ratio and root consumption by insects. This increase in herbivore damage may make *M. stipoides* more susceptible to herbivory under predicted climate change than other competing species, with consequences for the composition of grassland systems. As hypothesised, we found that *M. stipoides* was a better quality host that *C. refractus*, and that eCO_2_ had a bigger impact on *M. stipoides*. Our findings also matched our predictions that herbivore performance on *M. stipoides* would be worse under eCO_2_ and compensatory feeding would take place, but we did not find that increased feeding compensated for this decrease in performance.

C_3_ and C_4_ grasses differ in physiological, anatomical and chemical traits which are thought to make C_3_ grasses more susceptible to herbivory than C_4_ grasses, giving rise to the C_3_-C_4_ hypothesis [27]. This states that herbivores should select and perform better on C_3_ rather than C_4_ plants because they find them easier and more nutritious to consume. Compared to C_3_ grasses, C_4_ grasses tend to be lower in protein, which is also less accessible to herbivores as it is stored in bundle sheath cells, and possess higher levels of structural carbohydrates, making them less suitable host plants for folivores [27], [28]. The lower demands and uptake of N by C_4_ grasses compared to C_3_ grasses may similarly result in lower root N concentrations in C_4_ grasses and make them less favourable for root herbivores [26]. Under aCO_2_, *M. stipoides* represented a superior host for root herbivores than the C_4_ grass *C. refractus*, in line with the C_3_-C_4_ hypothesis [27]. While tests with further species are needed, these findings provide some initial support for the C_3_-C_4_ hypothesis potentially operating belowground as well as aboveground.

Despite evidence for compensatory feeding on the *M. stipoides* at eCO_2_, insect performance remained depressed at eCO_2_, suggesting that increased levels of herbivory were not enough to compensate for reduced plant quality. Similar effects occur for aboveground herbivores; Stiling and Cornelissen [2] concluded that most insect herbivores were generally unable to redress the problem of reduced food quality, and their abundance typically fell by 21% under eCO_2_. Compensatory feeding imposes extra energy requirements [6] which may be particularly demanding for soil-dwelling herbivores that have to physically burrow through the soil to access new root tissue [29]. Further work is needed to understand mechanisms of compensatory feeding for belowground herbivores, but the fact that they could not adequately compensate for deterioration in food quality suggests that energy constraints and thresholds for host plant quality may play a role. For example, the costs and benefits associated with compensatory feeding are likely to vary with both host quality and the impact of changes in quality on herbivore development and may only be possible above a quality threshold [30].

Compensatory feeding on C_3_ grasses might be particularly damaging since we observed no significant increases in plant biomass in response to eCO_2_. In their review, Hovenden and Williams [31] also note that Australian grasses are generally unresponsive to eCO_2_ in terms of growth, so they may be prone to higher herbivory levels without the advantage of enhanced growth rates seen in many other plants under eCO_2_. Our observations suggest that eCO_2_ may contribute to compositional changes in grass communities if C_3_ grasses are disproportionately damaged by root herbivores.
